# Mixed‐lineage leukaemia 1 contributes to endometrial stromal cells progesterone responsiveness during decidualization

**DOI:** 10.1111/jcmm.16030

**Published:** 2020-11-17

**Authors:** Yao Xiong, Yan Wang, Ling Ma, Ying Zhang, Xinlan Qu, Lei Huang, Xue Wen, Huimin Liu, Ming Zhang, Yuanzhen Zhang

**Affiliations:** ^1^ Reproductive Medicine Center Zhongnan Hospital of Wuhan University Wuhan China; ^2^ Hubei Clinical Research Center for Prenatal Diagnosis and Birth Health Wuhan China; ^3^ Department of Obstetrics and Gynecology Zhongnan Hospital of Wuhan University Wuhan China

**Keywords:** decidua, epigenetic regulation, implantation failure, MLL1, PGR

## Abstract

Studies have reported that non‐receptive endometrium or abnormal decidualization was closely related to recurrent implantation failure (RIF). MLL1 is a histone H3 lysine 4 trimethylation (H3K4me3) transferase that regulates the transcriptional activation of target genes. The role of MLL1 has been underexplored during decidualization. In our research, we found the expression of MLL1 was closely related to endometrial receptivity, and it was responsible to hormone stimulation. Inhibiting the function of MLL1 by MM102 reduced the transformation of HESCs. Furthermore, down‐regulation of MLL1 by siRNA transfection significantly decreased PGR and its target genes expression. MLL1 act as a co‐activator of ERα, and both of them were recruited to PGR regulatory regions, thus promote PGR transcription. Our study showed that MLL1 plays a key role in promoting progesterone signalling transmission.

## 1 INTRODUCTION

Assisted reproductive technologies have helped countless women to conceive healthy offspring; however, the embryo implantation success rate is still low. Repeated implantation failure (RIF) is an unsolved and challenging technical problem. Although the definition of RIF is not clear, most reproductive medicine centres defining RIF as failure to get pregnant in at least three or more embryo implantation cycles, and one or two embryos of high‐grade quality are transferred in each cycle.^1^ Abnormal endometrial receptivity is the major cause of RIF.^2^ During human menstrual cycle, the endometrium undergoes structural and functional changes, with endometrial stromal fibroblasts differentiating into decidualized cells that produce various cytokines and secreted proteins essential for embryo implantation.^3^ In RIF patients, aberrant gene expression has been observed during secretory phase.^4^, ^5^ However, the precise mechanism of abnormal decidualization of RIF has not been elucidated. Furthermore, increasing evidence indicates that hormones can affect the histone modification of genes during the menstrual cycle, which can inadvertently affect endometrial function.^6^, ^7^, ^8^ Histone modifications regulate gene expression by altering chromosomal structure and transcriptional activity. When histone modification enzyme complexes act on chromatin, they loosen up the structure of chromatin, making it accessible to transcription factors, which enabling genes transcription. Thereby inducing structural and functional endometrial changes during the menstrual cycle.^9^

H3K4 methylation in mammalian cells is modified by multiple methyltransferases, including MLL1‐4, SET1A and SET1B. MLL1 gene (also known as KMT2A) has been intensively studied as its crucial roles in hematopoiesis and embryo development^10^, ^11^ MLL1 regulates histone H3 lysine 4 methyltransferase activity, this kind of chromatin modification required for epigenetic transcriptional activation.^12^ It was reported MLL1 plays crucial role in cellular proliferation and differentiation.^13^, ^14^ Dysfunction of it may lead to aberrant stem cell development.^15^ MLL1 is also responsible for critical chromosomal rearrangements that underlie acute lymphoid leukaemia, and it is a transcriptional regulator of key target genes, including many *HOX* genes.^16^ In HeLa cells, MLL1 modulates the H3K4 trimethylation level of *HOX* genes (ie *HOXA5*, *HOXA7*, *HOXA10*) promoter region and affects the cell cycle by regulating *HOX* genes expression.^17^ Interestingly, *HOXA10* is an indispensable gene for the decidualization in endometrial stromal cells.^18^ Abnormal HOXA10 expression during the luteal phase usually associates with endometriosis, RIF and miscarriage.^5^, ^19^ Although MLL1 is a key regulator of *HOX*10, the role of MLL1 hormonal regulation of *HOXA10* gene during endometrial decidualization and embryo implantation has been underexplored.

It was demonstrated that MLL1 has one nuclear receptor box domain, which makes it plays a critical role in steroid hormone–mediated gene activation and signalling.^13^ Endometrium decidualization is a transformation process highly regulated by steroid hormone, progesterone is the primary driver of this transformation process. We suspect that hormonal fluctuations during decidualization would affect the expression of MLL1. In our research, we detected the expression of MLL1 in vivo and in vitro during decidualization. We also performed experiments to explore the function of MLL1 in decidualization.

## 2 MATERIALS AND METHODS

### 2.1 Ethical approval

The collection of human endometrial tissues was approved by Medical Ethics Committee, Zhongnan Hospital of Wuhan University (Approval No. 2017061). The endometrium tissues were collected only after written informed consent from patients.

### 2.2 Human endometrium samples

Patients were recruited from the department of Reproductive Medicine Center, Zhongnan Hospital of Wuhan University. The patients who were <40 years old, have normal ovarian reserve (follicle‐stimulating hormone <10 mIU/mL on day 2‐3 of menstrual cycle). But women with endometriosis, intrauterine adhesion, endometrial polyps, hydrosalpinx, submucous myomas and atrophic endometrium (<5.5 mm) were excluded. The endometrial tissues were obtained with a Pipelle catheter (GuardKing), secretory endometrial biopsies were normally performed either on 7 days after LH peak in natural menstrual cycle, or 5 days after taking progesterone in hormone replacement therapy (HRT) cycle, 26 secretory endometrial tissues were taken from 10 normal women and 16 RIF patients. The proliferative endometrial tissues were collected after 2 days of menstruation, 20 proliferative endometrial tissues were observed from 11 normal women and nine RIF women. Women who were failure to achieve clinical pregnancy after transfer of at least four good quality embryos in a minimum of three cycles defined as RIF (n = 25). Infertility caused by male factors, or women became pregnant less than two cycles after in vitro fertilization were included in the control group (n = 21). For primary stromal cells culture, endometrial tissues were obtained from another six normal women. Tissue samples processed immediately for primary cell culture or frozen in liquid nitrogen for RNA extraction. Tissue samples for immunohistochemistry (IHC) analysis were immediately placed in 10% formalin and embedded in paraffin.

### 2.3 Immunohistochemistry analysis

Immunohistochemistry staining was performed on paraffin sections with antibody against MLL1 (1:200; Abcam) and antibody against HOXA10 (1:200; Santa cruz). Immunostaining was performed as previous research described. Endometrial tissues were fixed by 4% paraformaldehyde and embedded in paraffin. Then the sections were deparaffinized and rehydrated in graded ethanol, and antigen retrieval was performed. Sections were then treated with 3% hydrogen peroxide for 5 minutes to inhibit endogenous peroxidase activity. After blocking for 30 minutes, sections were incubated overnight at 4°C with primary antibodies. On the next day, sections were incubated with peroxidase‐labelled anti‐rabbit IgG for 30 minutes. Finally, all slides were incubated with DAB‐Substrate (Beyotime) and counterstained in haematoxylin before they were dehydrated and mounted. IgG antibody was used in human endometrium as a negative control (Data were not shown). Ten fields were selected for each immunohistochemical section, and all these slides were used to conduct semi‐quantitative histologic scoring (H‐score) analysis by ImageJ.

### 2.4 Human endometrial stromal cells culture and in vitro decidualization

Fresh endometrial tissues were mixed and washed in PBS several times for removing traces of blood, then the tissues were cut into 1 mm^3^ sized pieces, followed by digesting with type II collagenase for 1 hour. Digested tissue mixture was filtered through a 150 μm pore size nylon meshes to separate mucus and undigested tissues. Then the mixed cells liquid was then filtered through a 38 μm pore size nylon meshes to extract endometrial stromal cells. The purified stromal cells were inoculated in DMEM/F12 medium containing 10% charcoal‐stripped FBS (CS‐FBS, Biological Industries) and antibiotics. To induce decidualization, the cell culture medium was changed to differential medium (DMEM/F12 with 2% CS‐FBS), containing 1 μM Medroxyprogesterone (MPA, Sigma), and 0.5 mM cyclic adenosine monophosphate (cAMP; Sigma). The medium was changed every 48 hours. Immortalized Human Endometrial Stromal Cells line (HESCs) was purchased from the American Type Culture Collection (ATCC Cat#CRL‐4003), the cells line culture medium was used in the same way as the primary cultured endometrial cells. As the enzymatic activity of MLL1 alone was weak, the H3K4 methyltransferase activity is dependent on the interaction with WDR5, RbBP5 and ASH2L. MM102 was reported to inhibit WDR5/MLL1 protein‐protein interaction, and it significantly reduced MLL1 targeting genes (*HoxA9 and Meis‐1*) in 25 and 50 µM.^20^ We used MM102 (Selleckchem) to inhibit MLL1 activity. MM102 powder was dissolved in dimethyl sulphoxide (DMSO).

### 2.5 Cells transfection

In order to ensure the transfection efficiency, immortalized HESCs were used for transfection. Lipofectamine 3000 Transfection Kit (Thermo) was used according to manufacturer's instructions. For MLL1 knockdown, MLL1 siRNA(50 nM) (RiboBio Co., Ltd.) was transfected as experimental group, the corresponding negative control siRNA (RiboBio Co., Ltd.) were transfected as control group. After 24h of transfection, cells were treated with cAMP + MPA for 0 or 4 days.

### 2.6 RNA extraction and real‐time quantitative PCR

Total RNA was extracted from cultured cells or tissues using RNA extraction kit (Aidlab Biotechnologies Co., Ltd), the operation method of RNA extraction was according to the manufacturer's procedure. 1 µg RNA from each sample was used to synthesize cDNA using the HisScript II Q RT SuperMix for qPCR (Vazyme) according to the manufacturer's methods. Quantitative real‐time PCR (qPCR) was performed on CFX Connect Real‐time PCR system using a kit of ChamQ™ SYBR qPCR Master Mix (Vazyme). The primer sequences for qPCR are shown in Table 1. The expression levels of mRNA were normalized to GAPDH and calculated using the 2^−ΔΔ^ *^C^* ^t^ method. All measurements were performed at least three times of independent experiments for each experimental condition.

**Table 1:** Primers used in RT‐qPCR

Primers for RT‐qPCR	
MLL1	5ʹ‐AACGGTTTCAGCTGCCTCTA‐3ʹ
	5ʹ‐TTTGGGTCACCTGAACTTCC‐3ʹ
PGR	5ʹ‐GTGCCTATCCTGCCTCTCAA‐3ʹ
	5ʹ‐GCCTTCCTCCTCCTCCTTTA‐3ʹ
HOXA10	5ʹ‐CCCTACACGAAGCACCAGACACT‐3ʹ
	5ʹ‐GCGTCGCCTGGAGATTCATC‐3ʹ
FOXO1	5ʹ‐GCCAAACTCACTACACCATA‐3ʹ
	5ʹ‐ACCAAAAACACACACAAATA‐3ʹ
STAT3	5ʹ‐CTTTTGTCAGCGATGGAGTA‐3ʹ
	5ʹ‐TGTTGACGGGTCTGAAGTTG‐3ʹ
Hand2	5ʹ‐ACCAGCTACATCGCCTACCT‐3ʹ
	5ʹ‐CTGCTCACTGTGCTTTTCAA‐3ʹ
PRL	5ʹ‐TCTCGCCTTTCTGCTTATTATAACC‐3ʹ
	5ʹ‐GATTCGGCACTTCAGGAGCTT‐3ʹ
IGFBP‐1	5ʹ‐CTATGATGGCTCGAAGGCTC‐3ʹ
	5ʹ‐TTCTTGTTGCAGTTTGGCAG‐3ʹ
GAPDH	5ʹ‐TCAGGCGTCTGTAGAGGCTT‐3ʹ
	5ʹ‐ATGCACATCCTTCGATAAGACTG‐3ʹ

### 2.7 Western blot

Total cell proteins were extracted using RIPA lysis buffer (Beyotime). The protein concentration of each sample was measured using Enhanced BCA Protein Assay Kit (Beyotime). 30 μg was taken from each sample for electrophoresis in 10% SDS‐PAGE gels, the separated proteins in gels were transferred to polyvinyl difluoride membranes, which was then blocked with QuikBlock Blocking Buffer for 15‐30 minutes (Beyotime). After that, the membranes were separately incubated with rabbit anti‐MLL1 antibody (dilution 1:1000; US Biological), mouse anti‐HOXA10 antibody (dilution 1:500; Santa Cruz biotechnology), rabbit anti‐Histone H3 (tri‐methyl K4) antibody (dilution 1:1000; abcam), rabbit anti‐PGR antibody (dilution 1:1000; CST), mouse anti‐β‐actin antibody(dilution 1:6000; OriGene) and mouse anti‐GAPDH (dilution 1:6000; Proteintech) overnight at 4°C. On the second day, the membranes were washed with PBS and then incubated with the peroxidase‐conjugated second antibody for 1 hour at room temperature. After washing again with PBS, membranes were visualized by ECL system (Tanon, Shanghai) using the High sensitivity ECL chemiluminescence detection kit (Vazyme, China). Image J was used for semi‐quantitative analysis of protein expression.

### 2.8 Co‐Immunoprecipitation (Co‐IP)

Cells before or after decidualization were lysed in 1 mL lysis buffer, equal amounts of proteins were used for immunoprecipitation. IP was performed using immunoprecipitation kit (Sangon Biotech) following the manufacturer's instructions. Appropriate amounts of antibody against MLL1, non‐specific IgG were then added and incubated overnight at 4°C. Then, the mixture liquids were added to 18 μL protein A‐sepharose beads for incubation overnight at 4°C. After washing several times, the bound proteins were eluted and separated by SDS‐PAGE. Antibody used for immunoblotting were rabbit antibody against ERα (dilution 1:1000; abcam), rabbit antibody MLL1‐c (dilution 1:1000; bethyl laboratory, A300‐374A).

### 2.9 ChIP‐qPCR

As the large number of cells required, HESCs were used for ChIP‐qPCR experiments, the cells were cross‐linked, lysed and sheared by sonication following protocols of Simple ChIP Plus Sonication Chromatin IP Kit from CST (#56383). Make sure average length of ultrasound‐interrupted DNA was 0‐1 kb, evaluated by agarose gel electrophoresis. Keep 2% of the chromatin fragments storing at −20°C, waiting to be used later as input groups for normalization. In each immunoprecipitation (IP) reaction, 4 μg of DNA was taken to separately incubate overnight with 10 μL antibodies against Tri‐Methyl‐Histone H3 (Lys4) Antibody (CST, #9727), 5 μL antibody against MLL1(GeneText, #GTX17959), 1 μL ERα antibody (abcam, #ab32063) and 1 μL antibodies against IgG (CST, #2729). The cross‐linking of IP and Input were reversed at 65°C for 2 hours, the DNA was then purified according to the instructions. The sequences of primers used in ChIP‐qPCR were as follows: PGR promoter region, 5ʹ‐GCTCCCCACTTGCCGCTCGCTG‐3ʹ(forward), 5ʹ‐TCGGGAATATAGGGGCAGAGGGAGGAGAA‐3ʹ(reverse); PGR enhancer 1 region, 5′‐GCCTGACCTGTTGCTTCAAT‐3ʹ(forward), 5ʹ‐GCAGGACGACTTCTCAGACC‐3ʹ(reverse); PGR enhancer 2 region, 5ʹ‐AACGTGTTTGCATCTTGCTG‐3ʹ(forward), 5ʹ‐GGGCTGGCTTTTATCATTCA‐3ʹ(reverse).

### 2.10 Statistical analysis

The GraphPad Prism 7 program (GraphPad Software Inc) was used for statistical analysis. All values are shown as the mean ± SD. Comparison between two groups were performed with Student's *t* test, differences among multiple groups were evaluated by one‐way ANOVA analysis followed by Tukey's multiple comparisons test. Correlations between HOXA10 and MLL1 staining H‐scores were analysed by means of the Pearson correlation. Statistical significance was defined as *P* < .05.

## 3 RESULTS

### 3.1 Decreased endometrial MLL1 and HOXA10 expression in women with RIF

**Figure 1:**
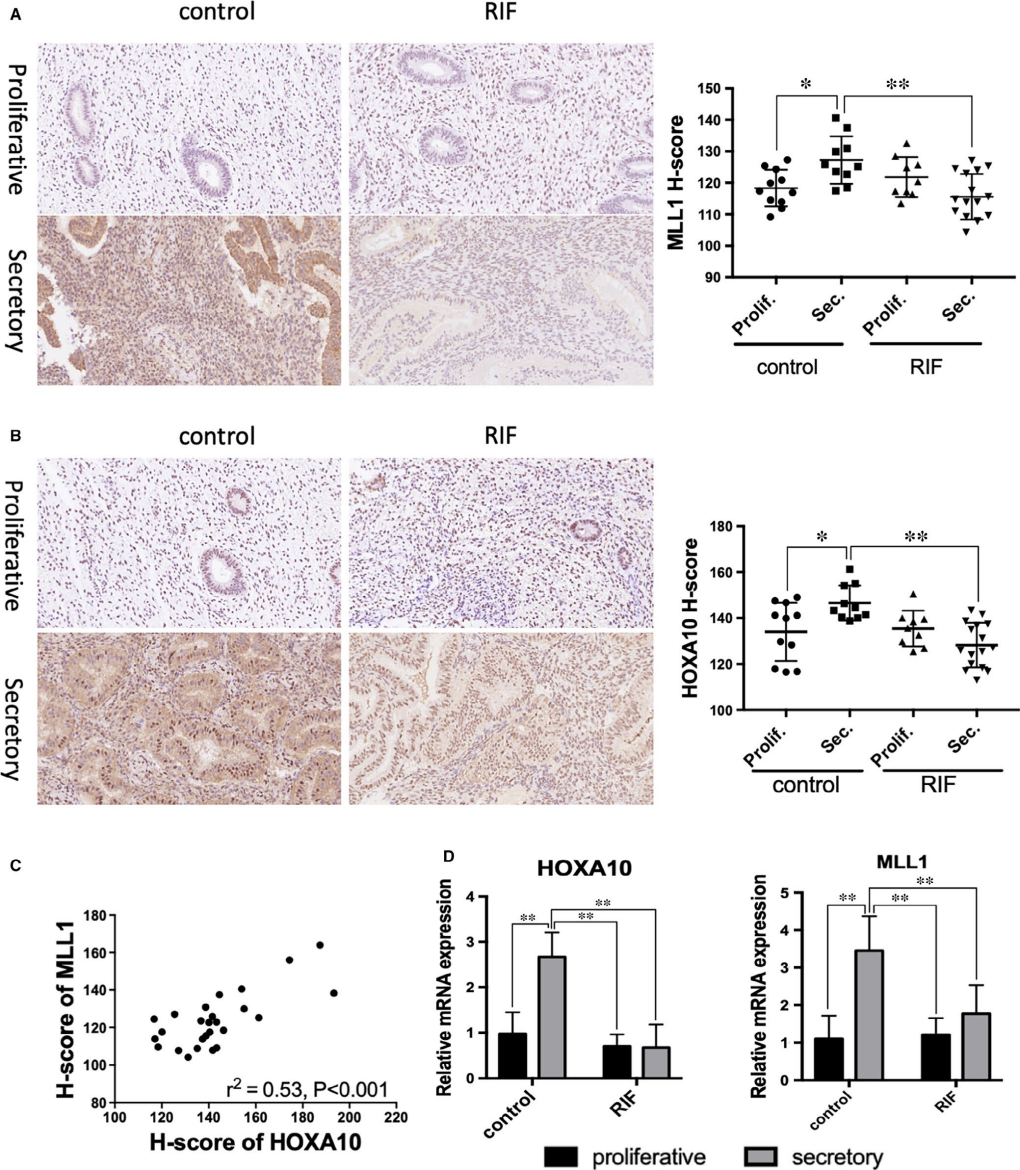
Endometrium HOXA10 and MLL1 expression in women with RIF and control. A, B, Immunohistochemical staining of endometrial MLL1 during proliferative and secretory phases in fertile women of control group and women with RIF. Photographs were taken at magnifications of 200×. C, Correlation analysis of HOXA10 and MLL1 expression during the secretory phase in control and RIF women. Immunohistochemical H‐scores of HOXA10 and MLL1 in endometrium from women with RIF and control group. D, HOXA10 and MLL1 mRNA levels analysis by RT‐qPCR in the different groups. RIF = repeated implantation failure, Prolif. = Proliferative, Sec. = Secretory. *P < .05, **P < .01

Endometrium MLL1 and HOXA10 proteins expression was examined during menstrual cycle. Both MLL1 and HOXA10 were expressed in the nucleus. As shown in Figure 1A, the intensity of the MLL1 nuclear signal was stronger at secretory phase than proliferative phase in control group. However, endometrial MLL1 expression in patients with RIF did not increase during the secretory phase, it was significantly lower compared to control group (*P* < .01, Figure 1A). There was no significant difference in the staining intensity was observed between the two groups during the proliferative phase (Figure 1A). The pattern of HOXA10 expression was similar to that of MLL1 (Figure 1B). In control group of fertile women, the HOXA10 H‐score was higher (*P* < .05) during the secretory phase than proliferative phase. But the average HOXA10 H‐score was lower (*P* < .01) in women with RIF than that in control group of fertile women during the secretory phase (Figure 1B). Correlation analysis revealed a significant positive correlation (*P* < .001) between MLL1 and HOXA10 H‐scores in secretory phase (Figure 1C). The mRNA expression of MLL1 and HOXA10 were detected by qPCR (Figure 1D). HOXA10 and MLL1 mRNA expression were higher in secretory phase of control groups. Both of MLL1 and HOXA10 mRNA expression during the secretory phase in women with RIF didn't increased significantly compared to proliferative phase, but they were reduced compared to control group in secretory phase (Figure 1D). These results indicate that MLL1 and HOXA10 loss in secretory phase may associated with RIF and suggest a potential role of MLL1 during endometrial decidualization.

### 3.2 Progesterone mediates MLL1 expression in endometrial stromal cells during in vitro decidualization

**Figure 2:**
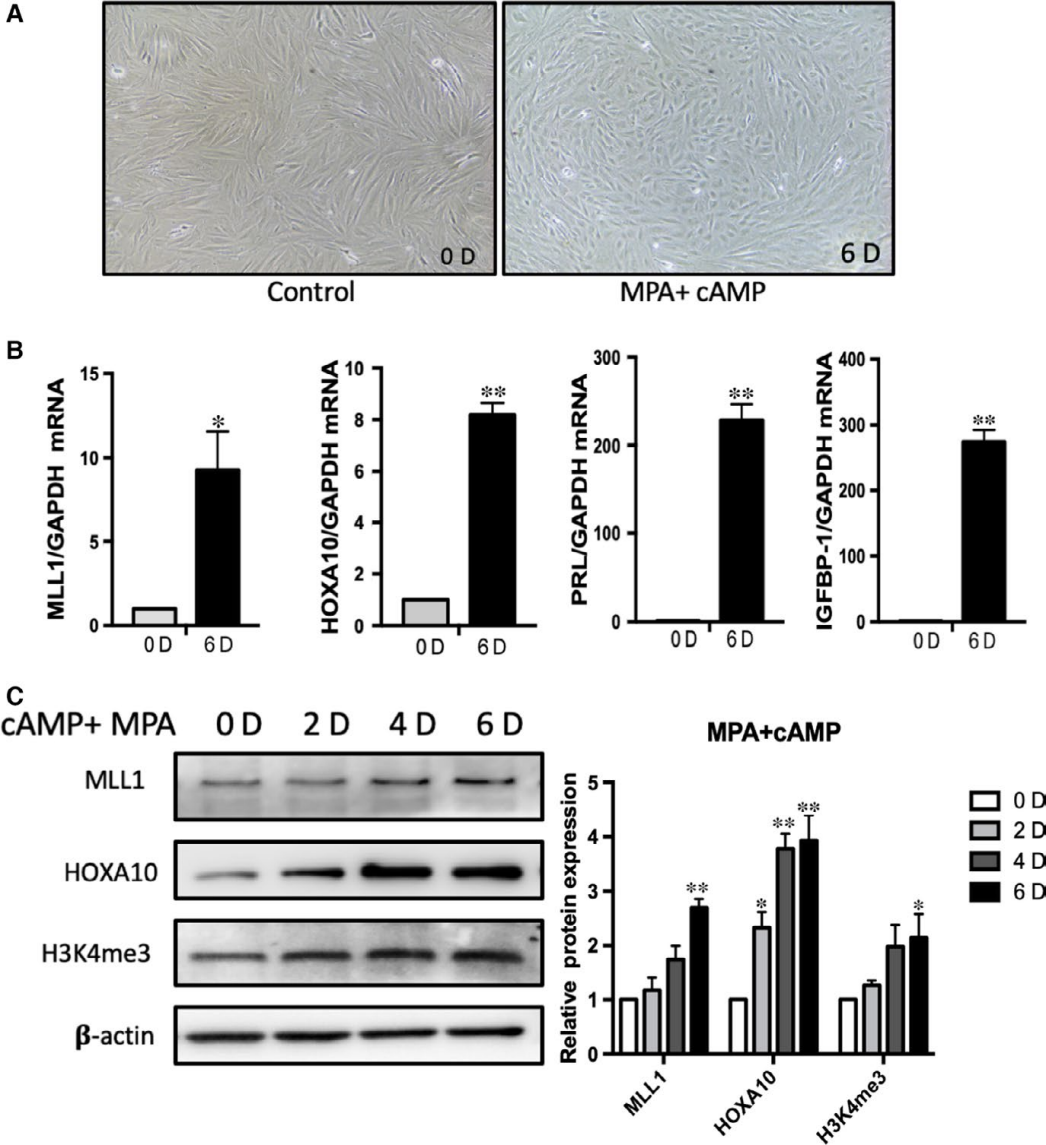
MLL1 and HOXA10 expression in primary cultured human endometrium stromal cells during in vitro decidualization. A, Morphological changes in primary cultured stromal cells before and after treatment with MPA + cAMP for 6 days. Photographs were taken at magnifications of 200×. B, RT‐qPCR analysis of MLL1, HOXA10, IGFBP‐1 and PRL mRNA levels after MPA + cAMP treatment for 6days. The values were normalized to the *GAPDH* level. Results are combined data from four experiments and each value is represented as mean ± SD. C, The proteins expression of MLL1, HOXA10, and H3K4me3 level in primary cultured stromal cells before and after treatment with MPA + cAMP for 2, 4 and 6 days. Represented data are from four independent experiments and each value is represented as mean ± SD. The values were normalized to the GAPDH expressions. *P < .05, **P < .01 vs 0D

Given the role of MLL1 in H3K4 trimethylation and the fact that histone tail modification can trigger gene activation, and MLL1 expression was related to decidualization, we investigated whether this modification was affected by the key hormones of decidua. Progesterone plays a major role in preparing the endometrium for embryo implantation, it acts by binding and activating PGR.^21^ cyclic adenosine monophosphate (cAMP) is another widely used decidualization stimulus to induce decidual markers in endometrial stromal cells. During decidualization, the intracellular cAMP level is significantly increased, activation of the cAMP pathway is essential for PGR regulating decidual gene networks, and the elevated intracellular cAMP levels are sustained by rising progesterone levels.^3^ Western blot and qPCR were carried out using primary cultured human endometrial stromal cells treated with medroxyprogesterone acetate (MPA) and cAMP in combination for 2, 4, 6 days. As shown in Figure 2A, the shape of stromal cells changed from fibroblastic appearance to round‐shaped morphologies upon combination treatment of MPA and cAMP on day 6. The mRNA levels of MLL1 and HOXA10 increased 9.3‐fold and 8.2‐fold separately after decidual treatment (Figure 2B). Up‐regulated mRNA expression of two well‐established decidual markers, PRL and IGFBP‐1, confirmed the stromal cells were effectively decidualized (Figure 2B). The protein expression of MLL1 and HOXA10, as well as the total H3K4me3 protein levels gradually increased in stromal cells upon treatment with MPA and cAMP, and both of them achieved the highest levels at day 6 (*P* < .01, Figure 2C). These results suggested that the expression of MLL1 and its mediated histone modification can respond to progesterone signalling.

### 3.3 Inhibiting MLL1 activity impairs endometrial decidualization

**Figure 3:**
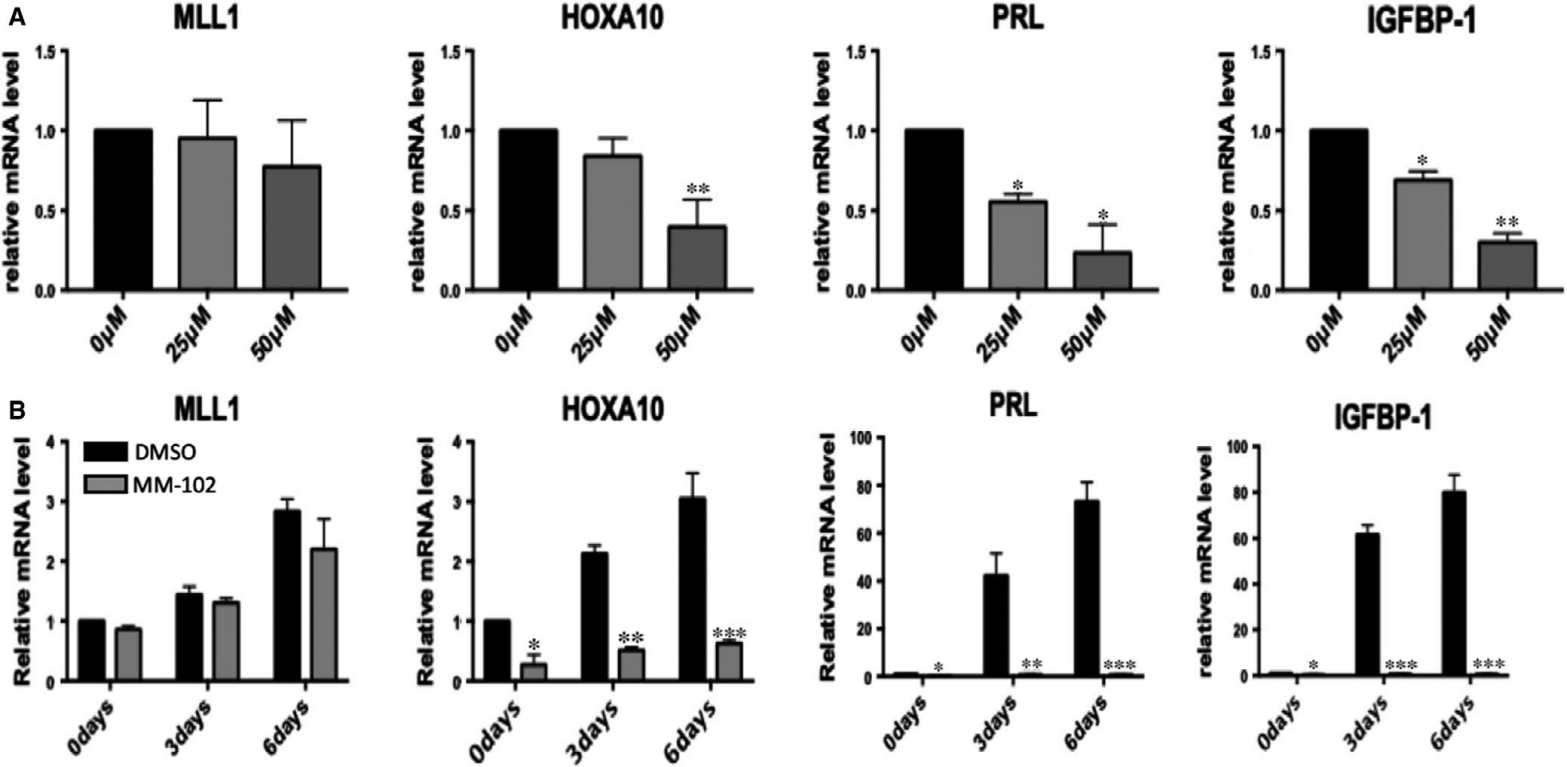
MM102 influence endometrial stromal cells decidualization. A, MLL1, HOXA10, PRL and IGFBP‐1 mRNA levels after treatment with 0, 25, and 50 µM of MM102 (n = 3, *P < .05, **P < .01 vs 0 µM). B, MLL1, HOXA10, PRL and IGFBP‐1 mRNA levels after treatment with 50 µM MM102 at decidualization days 0, 3 and 6. (n = 4, *P < .05, **P < .01, ***P < .001 vs DMSO)

We used a kind of MLL1 inhibitor MM102, to reduce the interaction between MLL1 and its co‐factors, thus inhibiting its transcriptional activation of downstream genes.^20^ After MM102 treatment, HOXA10 mRNA expression was significantly reduced in 50 μM, PRL and IGFBP‐1 were decreased in 25 and 50 µM without signs of massive cell death (Figure 3A and Figure S1). Therefore, 50 µM of MM102 was used in the subsequent time‐course experiments. In MM102 treated group, MLL1 did not significantly decreased, but HOXA10, PRL and IGFBP‐1 mRNA were persistent reduced compared to DMSO group during MPA + cAMP induced decidualization (Figure 3B).

### 3.4 Silencing of MLL1 prevents decidualization and affects progesterone signalling

**Figure 4:**
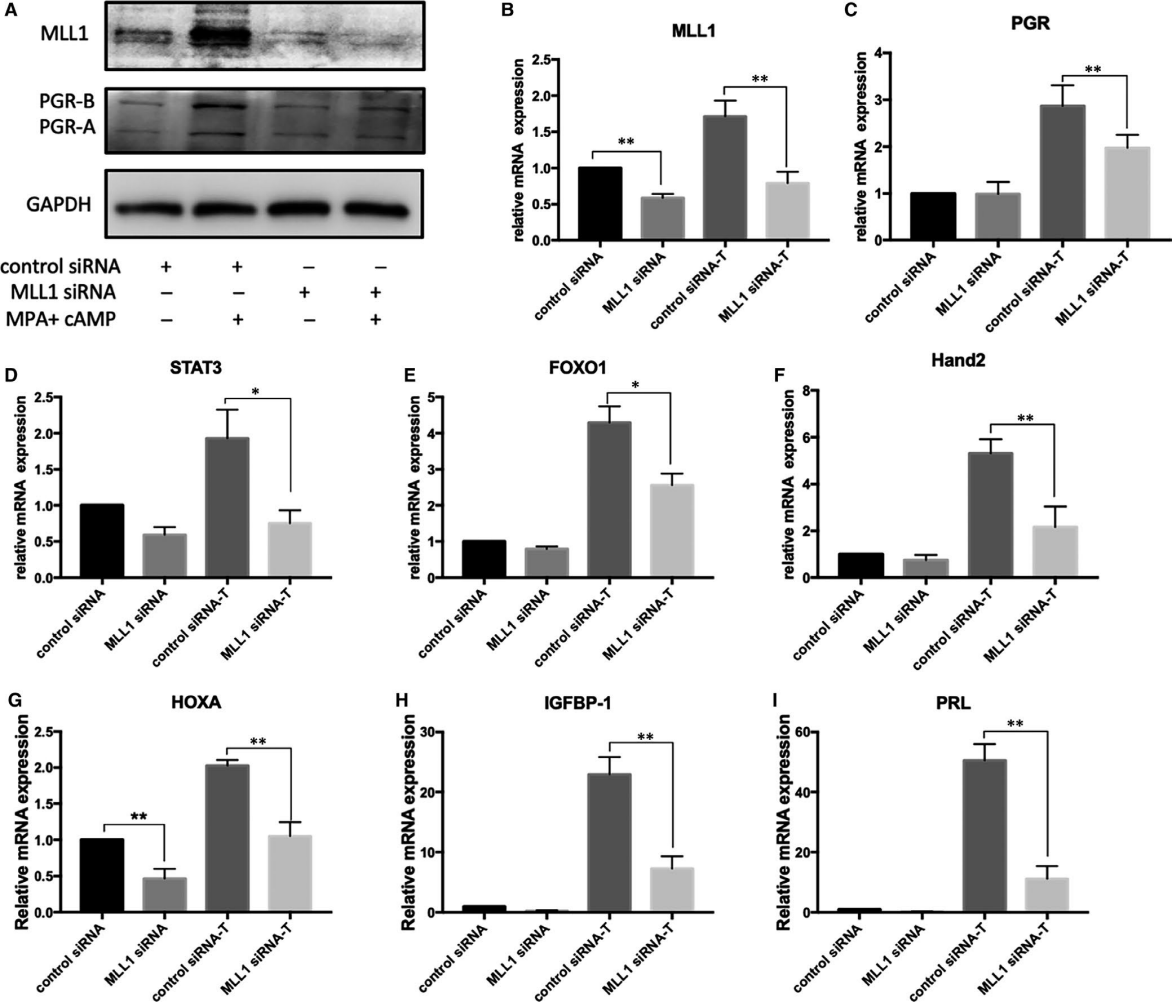
MLL1 is required for decidualization in HESCs. A, Western blot analysis of MLL1 and PGR protein levels in HESCs treated with control siRNA or MLL1 siRNA, followed by decidualization for 0 or 4 days. B‐I, RT‐qPCR analysis of MLL1, PGR, STAT3, FOXO1, Hand2, HOXA10, PRL and IGFBP‐1 levels in HESCs treated with control siRNA or MLL1 siRNA, followed by decidualization for 0 or 4 days. Results are represented as mean ± SD from three experiments with different cell preparations, *P < .05, **P < .01

To study the role of MLL1 in decidualization, siRNA targeting the *MLL1* gene was used to generate MLL1 knockdown in immortalized HESCs, then cells were cultured in the presence or absence of MPA + cAMP for 4 days to induce decidualization. The mRNA and protein levels of MLL1, PGR were higher after decidualization in cells treated with negative control siRNA (Figure 4A‐C). However, *MLL1* knockdown reversed the up‐regulated PGR levels upon decidualization of immortalized HESCs (Figure 4A,C). Knockdown of *MLL1* reduced decidual markers IGFBP‐1 and PRL mRNA expression (Figure 4H,I), the expression of PGR target gene STAT3, FOXO1, Hand2, HOXA10 mRNA expression were also inhibited (Figure 4D‐G). These results demonstrated that silencing of MLL1 affects progesterone signalling.

### 3.5 The recruitment of MLL1 and ERα at genomic target sites of PGR during HESCs

**Figure 5:**
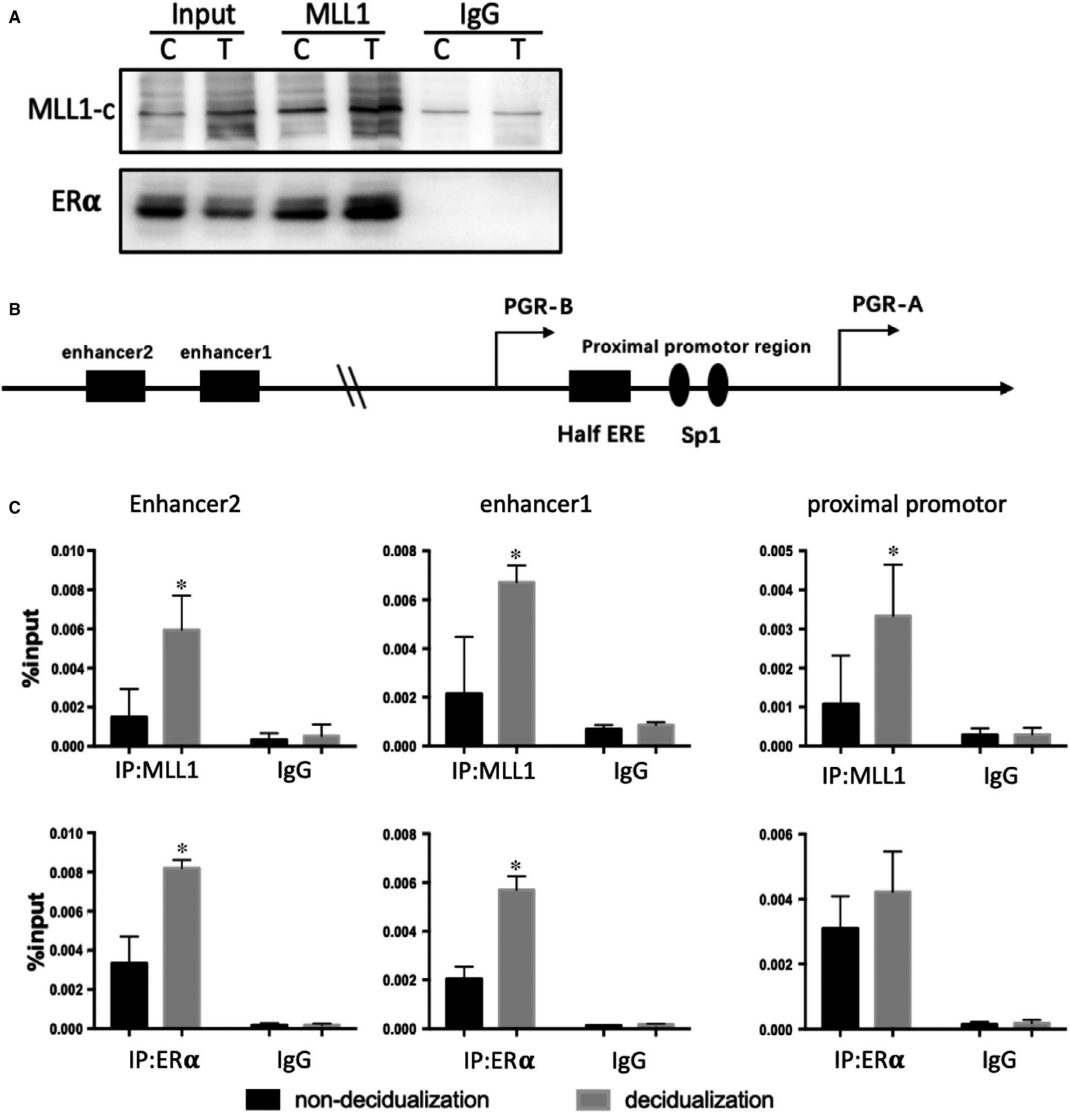
A, The co‐IP experiment of MLL1 and ERα were interacted during decidualization in HESCs. The picture shows the representative protein bands from the three experiments. B, Schematic diagrams of potential ERα binding sites in the PGR promoter and enhancer regions. C, CHIP‐qPCR was performed before and after MPA + cAMP treatment for 4 days, the amount of the indicated region of PGR gene precipitated by MLL1 and ERα antibodies were determined by qPCR. *P < .05

Because MLL1 was reported as a coregulator of ERα to activate target genes transcription, and ERα was essential for PGR expression, we surmised that MLL1 was a coregulator of ERα to activate PGR transcription. By using co‐IP, we found immunoprecipitation of MLL1 from HESCs extracts pulled down ERα before and after decidualization (Figure 5A). Furthermore, we observed that decidualization markedly enhanced the interaction between MLL1 and ERα (Figure 5A). These results predicted that MLL1 is a critical coregulatory factor recruited by ERα to activate PGR expression. There is a steroid hormone receptor binding sites of MLL1, this may be the loci where ERα binds to MLL1. PGR is a downstream target gene of ERα. During decidualization, silencing ERα can reduce the expression of PGR and inhibit decidual cells transition. In this study, we found that MLL1 can influence the expression of PGR, and MLL1 may be a coregulator of ERα, which activated PGR gene transcription. So, we used the CHIP‐qPCR technology to detect the recruitment of ERα and MLL1 in the combination of PGR gene regulatory regions. Although PGR was directly regulated by ERα, there were no consensus ERE (oestrogen response element) motifs near the transcription start sites of PGR. Researchers found that there were two ERα binding sites in enhancer region of PGR, located at −168 and −206 kb upstream of the PGR‐B TSS.^22^ Another binding site of ERα located at promoter A of PGR gene, which contains an ERE half site upstream of two adjacent Sp1 sites, the +571 ERE/Sp1 site.^23^ Here, we focused on the ERα binding sites located at −168 and −206 kb upstream from the PGR, and the proximal promoter region (+571 ERE/Sp1) (Figure 5B).

**Figure 6:**
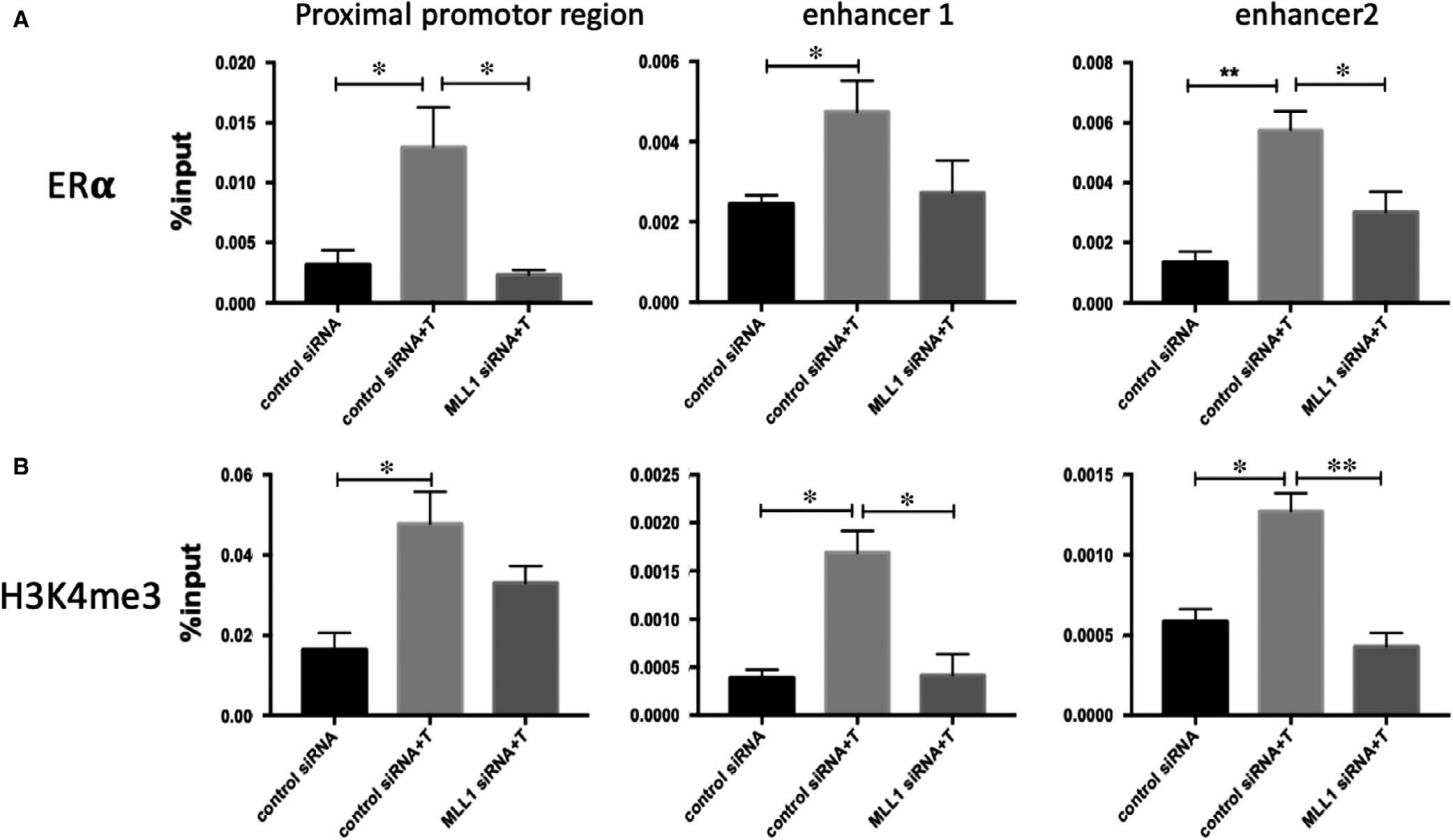
Effect of depletion of MLL1 on the H3K4me3 status and recruitment of ERα to PGR enhancer and promotor regions in HESCs. CHIP‐qPCR was performed after transfection with control siRNA, control siRNA followed by MPA + cAMP treatment and MLL1 siRNA followed by MPA + cAMP treatment. The amount of indicated region of PGR genes precipitated by H3K4me3 antibody or ERα determined by qPCR. *P < .05, **P < .01

In PGR gene, higher levels of MLL1 occupancy were observed at proximal promoter, and two enhancer regions of PGR (Figure 5C). Furthermore, ERα occupancy at these regions was also increased (Figure 5C). Depletion of MLL1 decreased the occupancy by ERα at PGR promoter region, enhancer1 and enhancer2 regions during decidualization (Figure 6A), and the H3K4me3 levels at these regions were also decreased (Figure 6B). MLL1 is an important regulator required for ERα recruitment on PGR and initiation of transcription of PGR gene.

## 4 DISCUSSION

How to improve the successful embryo implantation rate during IVF‐ET cycle is still a big problem for patients with repeated implantation failure. Progesterone supplementation alone cannot improve endometrial receptivity of some RIF patients. The pathogenesis of repeated transplantation failure is still unclear. It is very essential to explore the molecular mechanism of endometrial decidualization and endometrial receptivity.

Previous research has shown that increased H3K4me3 modification can activate the expression of many key target genes during decidualization,^24^ our study first explored the role of histone methyltransferase MLL1 during decidualization, which mainly mediated H3K4me3. Here, we found that endometrial HOXA10 and MLL1 expression were both increased during secretory phase in control group, but they all decreased in women with RIF compared with fertile women during the secretory phase. The expression of HOXA10 in uterine stromal cells is in a PGR‐dependent manner during decidualization^25^, ^26^, ^27^ with its level peaking during the secretory phase and then reducing after embryo implantation.^28^ We found MLL1 expression was positively correlated with HOXA10 expression during luteal phase. It was demonstrated that histone octamers near the *HOXA10* promoter region are extensively trimethylated at Lys4 of histone H3.^29^ MLL1 occupies much more extensive domain within a transcriptionally active region of the *HOXA* cluster than other genes.^30^ This maybe the reason why HOXA10 and MLL1 expression were positively correlated in human endometrial tissues. The results from in vivo human endometrium tissues suggest the possible involvement of MLL1 in decidualization.

MLL1, as a histone methyltransferase, mainly mediates the transcription of H3K4me3 modified activation gene.^13^ In the process of decidualization, the up‐regulation of a large number of gene expression may be related to MLL1. In recent years, the research on the mechanism of histone modification in endometrial decidualization has gradually increased. The decreased expression of deacetylase HDAC3 in eutopic endometrium in endometriosis patients directly affect the collagen gene expression. The expression of ER, PGR and their respective target genes in HDAC3 knockout mice were also decreased, which leads to abnormal decidualization in secretory phase.^26^ On the other hand, the expression of histone methyltransferase EZH2 decreased in the process of decidualization, thus weakened the modification of H3K27me3, and promoted the expression of PRL and IGFBP‐1.^31^ During decidualization, acetylation was highly expressed in the promoter or enhancer regions of PRL and IGFBP‐1.^6^ These histone modification mechanisms participated in the transformation of endometrial decidualization. Tamura et al^24^ found that H3K27ac and H3K4me3 modifications are involved in the up‐regulation of gene expression in the process of decidualization. As mentioned above, the role of H3K27ac modification in decidualization has been reported, but the role of H3K4me3 and MLL1 in decidualization have not been reported.

When we treated primary cultured human endometrial stromal cells with cAMP and MPA, we found that the expression of MLL1 increased, and with the increase of total H3K4me3 expression. MLL1 itself has only weak histone methyltransferase activity, and MLL1 needs to combine with three basic co‐regulatory factors WDR5, RBBP5 and ASH2L to fully play its histone modification function.^32^ MM‐102 is an inhibitor of MLL1, which affects the histone methyltransferase function of MLL1 by inhibiting the combination of MLL1 with its co‐active factors WDR5, ASH2L and RBBP5.^20^ The addition of MM‐102 in the process of decidualization inhibits the activity of MLL1, resulting the decreased decidual marker genes HOXA10, PRL and IGFBP‐1 expression. Therefore, MLL1 was involved in the transcriptional activation of decidualization‐related genes, thus influenced the transformation of cell decidualization.

Progesterone signalling pathway is the prerequisite regulation of decidualization. We further silenced the expression of MLL1 gene with siRNA, detected the expression of progesterone receptor PGR and its target genes related to decidualization. The results showed that the expression of PGR and its target genes decreased after silencing MLL1. Artinger et al^33^ found that PGR gene was down‐regulated after knocking out MLL1 gene, but the research did not further verify the regulation of MLL1 on PGR gene.

**Figure 7:**
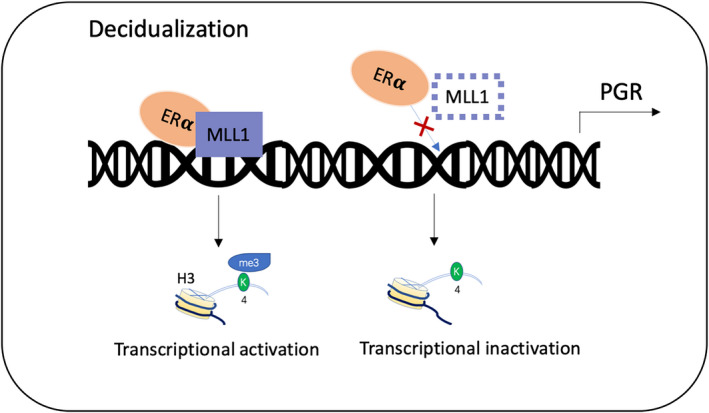
Picture showing how MLL1 interacts with ERα at PGR regulatory regions. MLL1 interacts with ERα, and promoting the H3K4me3 levels at the PGR regulatory regions. Depletion of MLL1 hampers the recruitment of ERα to PGR gene, and thus reduced the transcription of PGR

Our research further explored the mechanism of MLL1 on PGR gene. MLL1 was first known in acute myeloid leukaemia because of allelic fusion, which led to abnormal regulation of HOX family genes. However, MLL1 is not the only protein that regulates the transcription of HOXA10. ERα can act synergistically with MLL1 on the oestrogen response element region (ERE) of HOXA10 gene and regulate the transcription of HOXA10 gene.^34^ Our Co‐IP experiments showed that MLL1 and ERα interacting with each other in endometrial stromal cells, and the binding level of ERα and MLL1 increased after decidualization, so we speculated that MLL1 and ERα can jointly regulate their downstream target genes. The combination of MLL1 and ERα can not only promote the transcription of HOX gene, but also activate the target gene PGR.^35^ In breast cancer cells line MCF‐7, MLL1 and ERα were both recruited to PGR enhancer regions after oestrogen stimulation. Knockout of MLL1 significantly reduced the recruitment of ER α to PGR.^22^ Our CHIP‐qPCR experiment focused on the ERE/Sp1 site and two enhancer regions of PGR, we found the recruitment of MLL1 on the three potential binding sites were all increased after decidualization. The recruitment of ERα to PGR enhancer regions was also increased. While knocking out of MLL1 significantly decreased the enrichment of ERα at three binding sites, and the H3K4me3 levels were all deceased. The down‐regulated histone modification and reduced recruitment of ERα may affect PGR transcription. This result confirms our previous speculation that down‐regulation of MLL1 may affect PGR gene transcription by decreasing the recruitment of co‐active factor ERα to PGR gene (Figure 7).

Our study found that the expression of MLL1 was decreased in the endometrium of patients with RIF. In this case, even if progesterone was supplemented to patients, the abnormal expression of MLL1 may lead to reduced transcription of PGR, thus affecting progesterone signal. The weak reaction of endometrium to progesterone lead to progesterone resistance. So far, the aetiology of RIF is not clear, endometrial receptivity is the main factor, histone modification plays a key role in the establishment of endometrial receptivity.^24^ The expression of MLL1 in endometrium may be influenced by environmental factors. Our previous study found that BPA reduced the expression of MLL1 during decidualization and affect the histone modification of decidual marker genes in endometrial stromal cells.^36^ The abnormal expression of MLL1 caused by these external environmental factors will also affect progesterone signal transmission.

During decidualization, a highly complex network composed of immune cells and inflammatory mediators were involved.^37^ MLL1 was reported to regulate inflammatory genes and promote inflammatory environment. Inflammatory IL‐13 can up‐regulate MLL1 expression, as well as total H3K4me3 level in nasal epithelial cells, promoting CLCA1 and MUC5a transcription, and leading to formation of local inflammatory environment in nasal cavity.^38^ MLL1 mediated H3K4me3 modification was also involved in the transcriptional activation of inflammatory gene NF‐kB, and regulating the pro‐inflammatory response of macrophages.^39^ Whether MLL1 plays a role in the transcription regulation of inflammatory genes during decidualization needs further research.

In other endometrial receptivity disorders, abnormal MLL1‐mediated H3K4me3 modification in target gene promoter regions may also be involved. It was reported that the total methylation level of H3K4me3 in eutopic endometrium is higher than that in normal endometrium.^40^ But another research found that,^41^ the expression of MLL1 and total H3K4me3 level in eutopic and ectopic endometrium of patients with endometriosis were lower than that of normal endometrium. Samadieh et al^42^ examined the histone modification of HOXA10 in endometriosis, they found that there was no difference in H3K4me3 modification at HOXA10 promoter region in eutopic lesions in secretory phase compared with normal endometrium. Our recent research found that the MLL1 expression in the eutopic endometrium was decreased by using a mouse endometriosis model (the results were not published). Abnormal expression of MLL1 may result in decreased receptivity of eutopic endometrium in patients with endometriosis. In this study, the collected endometrial tissues from RIF patients, excluding chronic endometritis such as endometriosis, endometrial polyps and submucous myoma. But MLL1 may also abnormal expressed in these diseases, and affect endometrial receptivity.

A potential limitation of our research is that human endometrium tissue samples didn't include late proliferation, early and late secretory phases. Although we observed the higher expression of MLL1 in mid‐secretory phase, we couldn't see the more detailed changes of MLL1 with hormone fluctuation in menstrual cycle.

In conclusion, we first observed the increased expression of MLL1 was related to endometrial receptivity, then we focused on the biology role of MLL1 in endometrial decidualization. Through the induction of decidualization in vitro, we observed that MLL1 and the H3K4me3 levels increased. MLL1 act as a co‐acting factor of ERα, two of them were recruitment to the regulatory regions of PGR during decidualization. Down‐regulation of MLL1 affected the binding of ERα at PGR enhancer and promoter regions, thus inhibits PGR transcription. Given the results presented here, we speculated that MLL1 enables the transformation of HESCs to decidual cells, then generating a receptive endometrium for embryo implantation. By understanding the function of MLL1 in endometrial decidualization, it may help to develop related targeted therapy for RIF patients.

## CONFLICT OF INTEREST

The authors confirm that there are no conflicts.

## AUTHOR CONTRIBUTION

**Yao Xiong:** Data curation (lead); Project administration (lead); Writing‐original draft (lead). **Yan Wang:** Methodology (equal); Resources (equal). **Ling Ma:** Project administration (equal); Resources (equal). **Ying Zhang:** Investigation (equal); Methodology (equal). **Xinlan Qu:** Formal analysis (equal); Investigation (equal); Methodology (equal). **Lei Huang:** Methodology (equal); Project administration (equal). **Xue Wen:** Writing‐review & editing (equal). **Huimin Liu:** Formal analysis (equal). **Ming Zhang:** Conceptualization (lead); Writing‐review & editing (lead). **Yuanzhen Zhang:** Conceptualization (lead); Funding acquisition (lead); Resources (lead).

## Supporting information

Fig S1

Fig S2

## Data Availability

Data are available on request from the authors.
