# Supplementary figures and images for: Mixed‐lineage leukaemia 1 contributes to endometrial stromal cells progesterone responsiveness during decidualization
Source: J Cell Mol Med. 2020 Nov 17;25(1):297–308. doi: 10.1111/jcmm.16030 (PMC7810960; doi:10.1111/jcmm.16030)

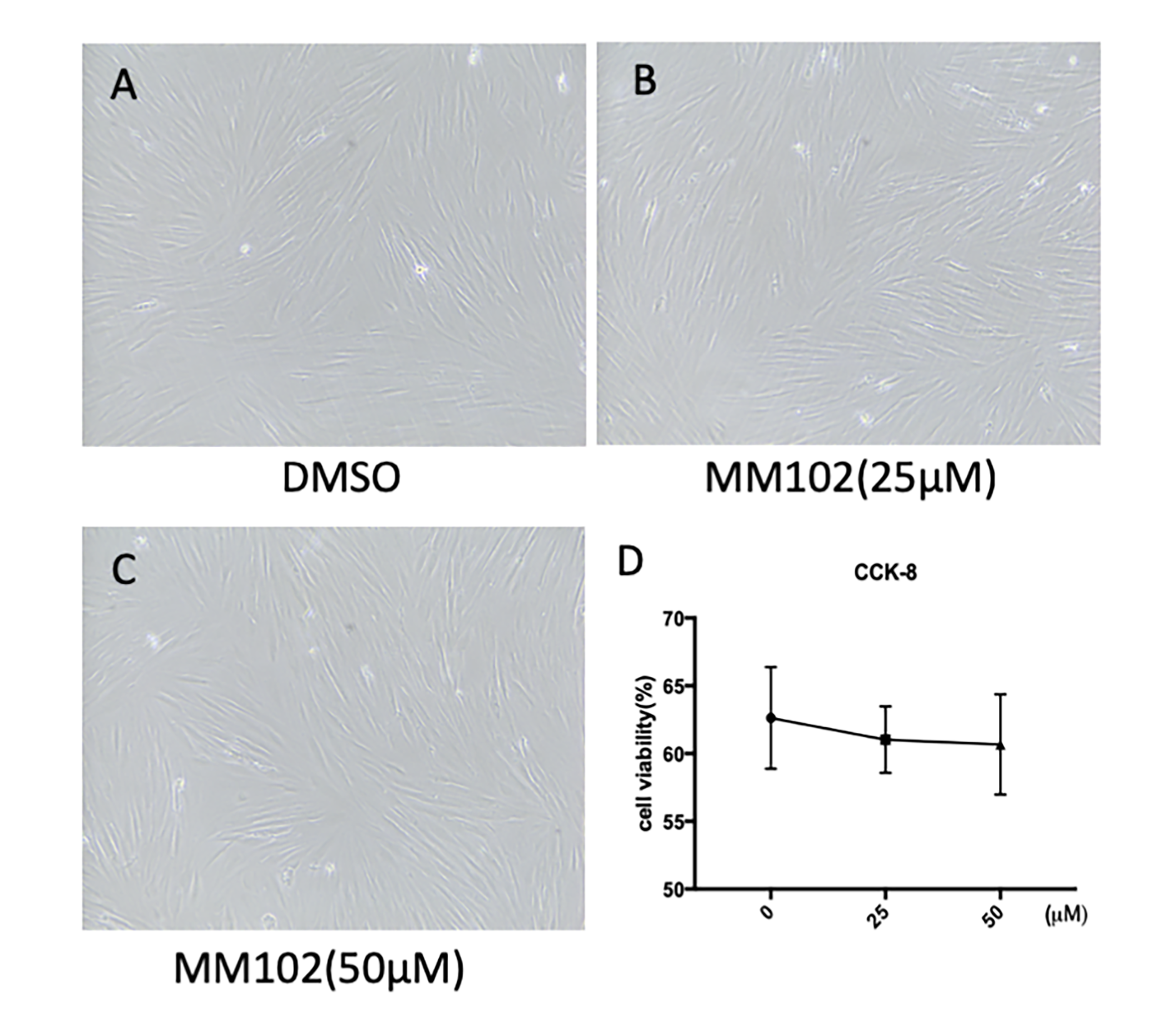

Supplement: Supplementary file 1 — Fig S1 [file JCMM-25-297-s001.tif]

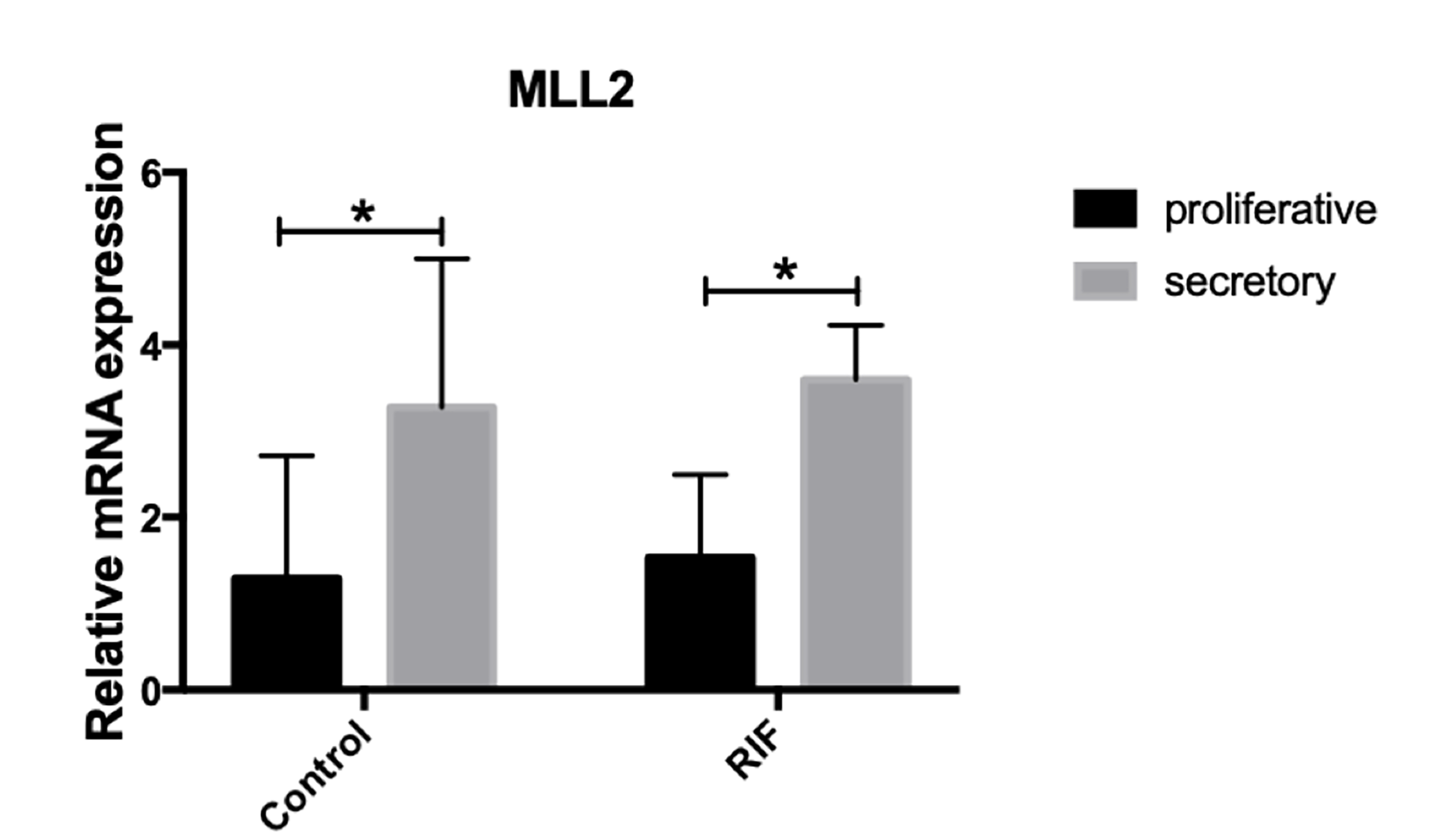

Supplement: Supplementary file 2 — Fig S2 [file JCMM-25-297-s002.tif]
